# Supplementary material for: A network dynamical simulation model for the study of antibiotic resistance in microbial communities
Source: Gut Microbes. 2026 Sep 19;18(1):2734703. doi: 10.1080/19490976.2026.2734703 (PMC13596919; doi:10.1080/19490976.2026.2734703)

Barabási−Albert networks: mean change in clustering coefficient (N = 1000)

Tiles and labels show mean ± SD across simulation replicates

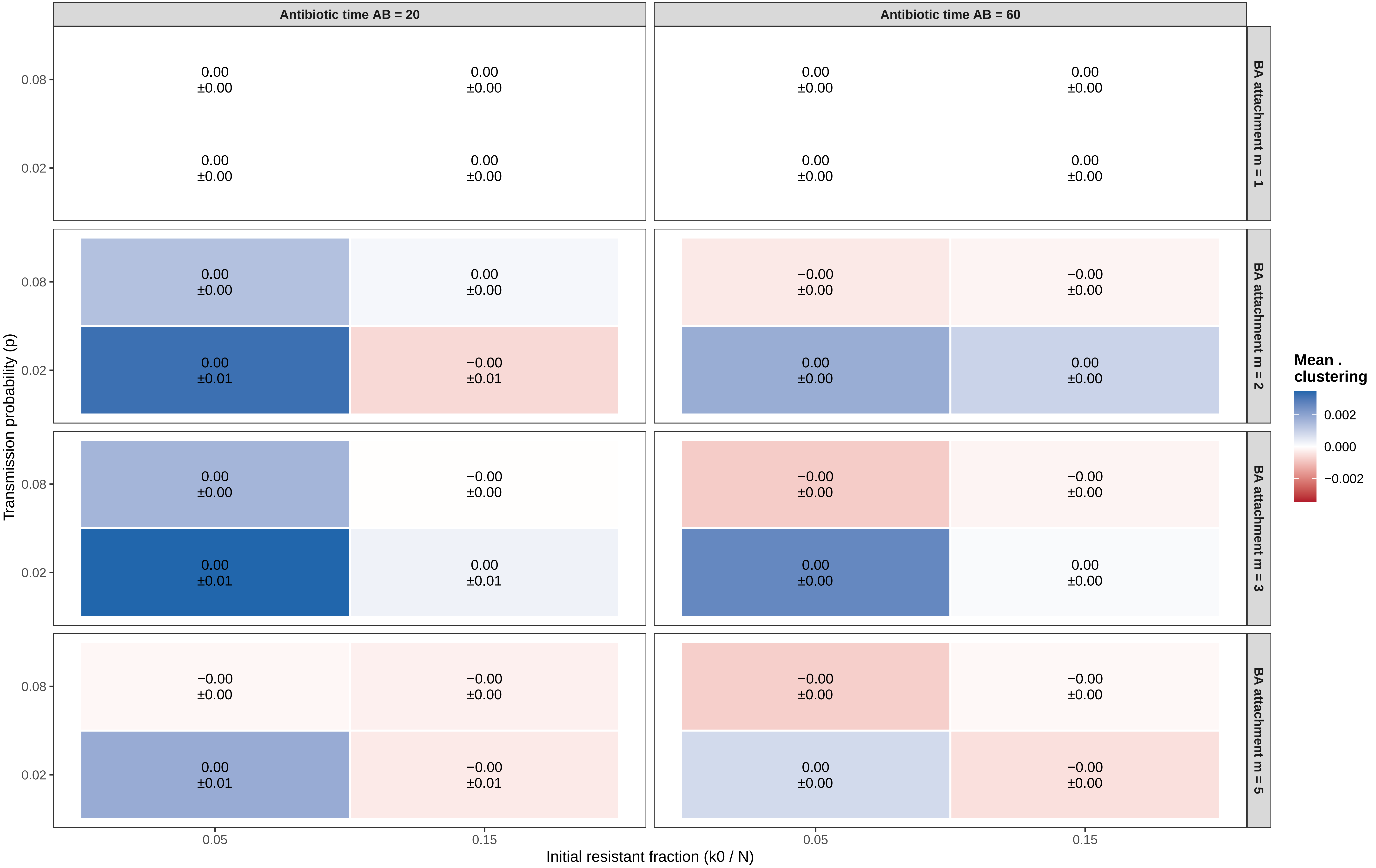

Supplement: Supplementary file 1 [file KGMI_A_2734703_SM9053.zip › supplementary_file_1/Figures/ba_delta_clustering_n1000.pdf]
